# Arthrokatadysis from post-injection gluteal muscular fibrosis case report

**DOI:** 10.1186/s12891-020-03766-5

**Published:** 2020-11-14

**Authors:** Yunfeng Mi, Biao Cheng

**Affiliations:** 1 Department of Orthopedics, Ningbo First Hospital, Affiliated Ningbo Hospital of Zhejiang University, No. 59 Liuting Street, Ningbo, Zhejiang Province 315010 China; 2 Department of Orthopedics, School of Medicine, Shanghai Tenth People’s Hospital, Tongji University School of Medicine, NO.301 Yanchang Middle Road, Shanghai, 200072 China

**Keywords:** Gluteal muscle contracture (GMC), Arthrokatadysis, Arthroscopic surgery, Case report

## Abstract

**Background:**

Gluteal muscle contracture (GMC) is a clinical syndrome characterized by the contracture of gluteal muscles, iliotibial band (ITB), and related fascia. GMC is much more prevalent in China, which has been proven to be associated with repeated intramuscular injections into the buttocks and the subsequent fibrosis and contracture.Generally, GMC is manifested mild. Here, we reported a severe case with arthrokatadysis.

**Case presentations:**

A 25-year old man received multiple intramuscular injections of penicillin in the buttock when he was diagnosed with acute tonsillitis at 6 years old. Since then, he was injected penicillin regularly in local hospital because of the repeated acute tonsillitis until he was in high school. When the patient was found by the physical education teacher to be running in a state of external rotation of both feet, he was suggested to go to the hospital for treatment and was initially diagnosed to have GMC. He complained of occasional pain and limited range of motion in the hip joints. X-ray showed a typical arthrokatadysis. After arthroscopic release of GMC, the patient recovered well.

**Conclusions:**

This is possibly the first reported case of arthrokatadysis that was caused by GMC after repeated intramuscular injections into the buttocks. Although the patient recovered well by arthroscopic surgical release of bilateral gluteus maximus contractures, GMC should be paid more attention and treated as early as possible.

## Background

Gluteal muscle contracture (GMC) is a clinical syndrome of gluteal muscle and fascia fiber degeneration and contracture caused by various factors, resulting in the characteristic gait and physical signs of hip joint with limited function [1].This disease was first reported by De Valderrama JAF in 1970 [2]. It is associated with the patient's congenital factors, scar constitution, immune factors, genetic factors, trauma factors, infection factors and surgical factors [3, 4]. Most GMCs are caused by repeated injections into the buttock [5]. Benzyl alcohol used as a dissolvent in intramuscular penicillin injections may elicit cell injury and fibrotic reparative tissue. Repeated intramuscular injections cause fibrosis secondary to hematoma, neuropathy, and ischemia [6]. A childhood incidence of 1–2.5% and a morbidity of 1.36% in affected individuals have been reported [7]. Patients often present with hip joints dysfunction and abnormal gait. Their knees can’t close together when they squat. Most patients with mild GMC have mild clinical symptoms. Nevertheless, we found a patient with arthrokatadysis due to severe GMC. This is possibly the first reported case of arthrokatadysis that was caused by GMC after repeated intramuscular injections into the buttocks.

## Case presentations

By age 6 years, the male patient began to receive multiple intramuscular injections of penicillin due to recurrent tonsillitis. By age 10 years, this type of therapy was administered for undetermined times. By age 12 years, the patient began to suffer from long-term walking bilateral buttocks pain on external snapping hip. At age 15 years, he could not close knees when he squatted and could not cross legs when he sat, either. He walked and ran with his feet in a state of external rotation. However, He had no obvious leg symptoms, low fever, night sweats, and numbness of both lower limbs. With GMC gradually developing, his hip joints were getting worse. He complained of a feeling of tugging at his hips and a limited motion in his hip joints.

### Clinical features

**Fig. 1:**
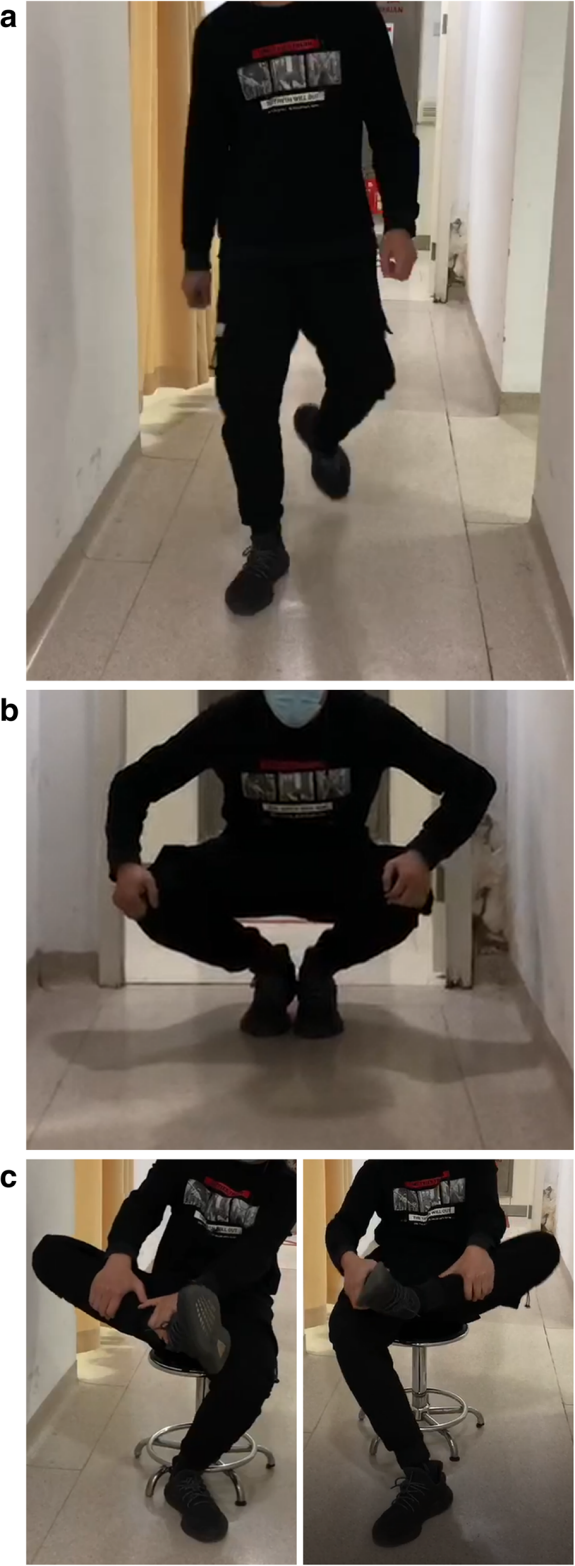
**a** The patient walks with his feet in external rotation. **b** The patient can’t keep his legs together when squatting. **c** The patient can’t cross his legs in the sitting position

At the patient’s first visit to hospital, he presented with an abnormal walking posture with both hips in an abducted, externally rotated position (Fig. 1a). He was unable to put his knees together in squatting position without losing balance (Fig. 1b). He couldn’t cross his legs in sitting position (Fig. 1c). There was restriction of flexion of both hips. A contracture band could be touched in the patient's buttocks. When doing hip joint flexion, he felt a sense of bounce at the femoral greater trochanter. Bilateral Ober signs were positive. However, no flattening and kyphotic deformity were observed in the patients' bilateral buttocks.

### Laboratory features

The patient had normal examination results for blood routine test, CRP (C-reactive protein), erythrocyte sedimentation rate, clotting function test, anti-streptococcal hemolysin "O" test, and rheumatoid factor test.

### Imaging features

**Fig. 2:**
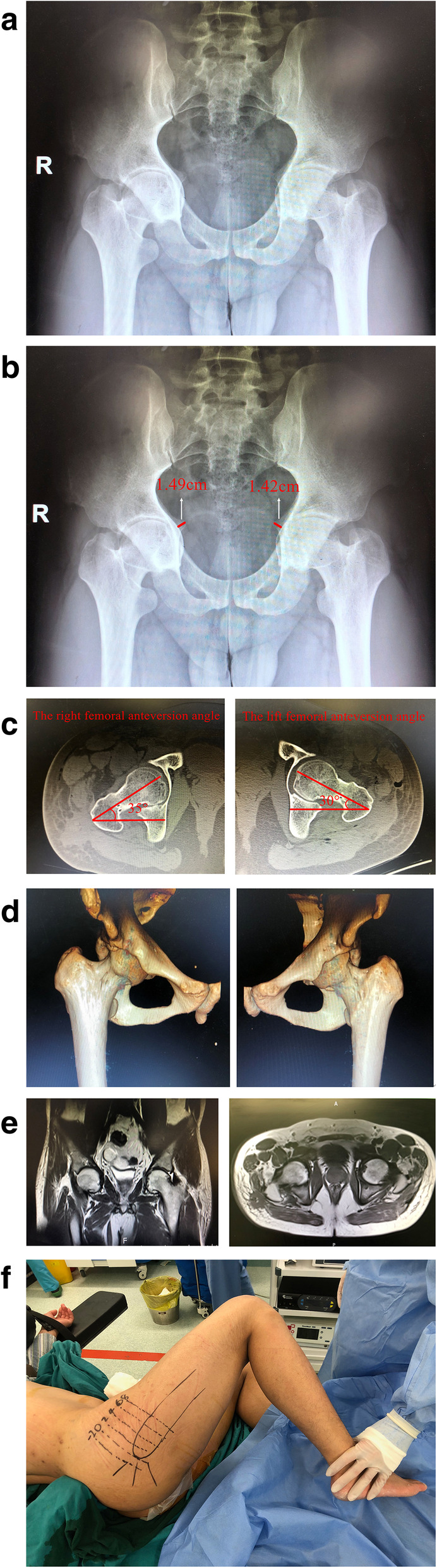
**a** The pelvic radiograph shows the inward depression of the hips with narrowing joint space. **b** A pelvic radiograph shows that the right femoral head and the left femoral head cave through the acetabular wall and exceeds Kohler's line by 1.49* cm* and 1.42 cm, respectively. **c**. The CT scan indicate that the hip joints is obviously sunken, the right femoral antetorsion is about 35 degrees, and the lift side is about 30 degrees. **d**. The three-dimensional CT shows the structure of the hip joints. **e**. The coronal and transverse magnetic resonance imaging indicate normal signals of femoral head and no signs of femoral head necrosis. **f** The surgical marker diagram illustrates that the vertex of the greater trochanter of the hip joint is denoted 0, parallel lines are made up and down, with an average interval of 2 cm between each line

An anteroposterior radiograph of the pelvis showed obvious bilateral hip joints depression (Fig. 2a), The vertical distance from the midpoint of the acetabular line (the medial wall of the acetabulum) to the iliac line (the side of the quadrilateral) was 1.49 cm to the right, and 1.42 cm to the left (Fig. 2b). The CT scan of the patient's hip joints showed the right femoral anteversion angle was 35 degrees, and the lift femoral anteversion angle was 30 degrees (Fig. 2c). The 3D image of the hip joints showed the bony structure (Fig. 2d). MRI showed no signs of femoral head necrosis and no significant atrophy of bilateral gluteus (Fig. 2e).

### Treatment

**Fig. 3:**
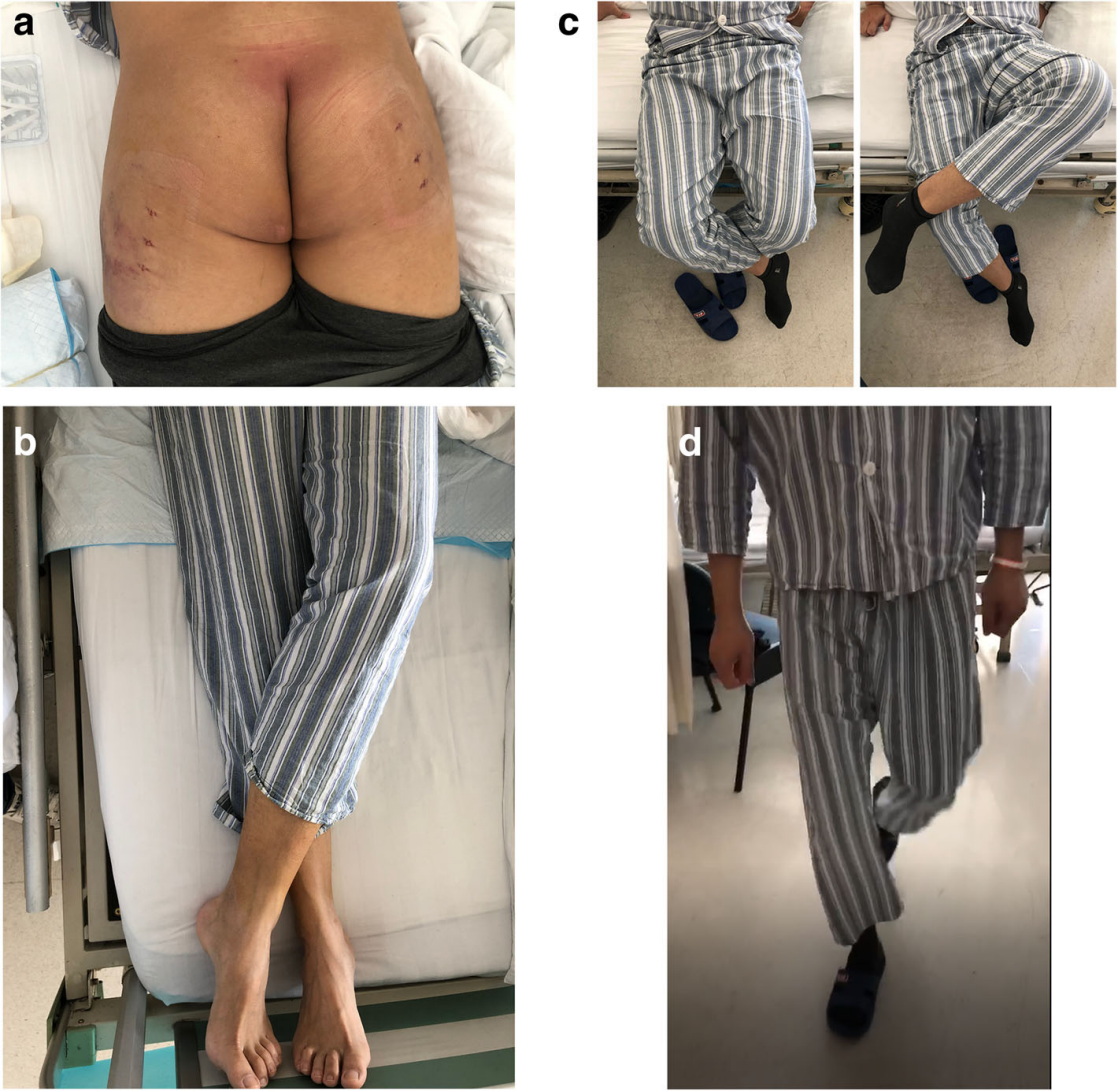
**a **A clinical photograph taken on the first day after the surgery shows two mini incision and mild swelling over the operation area. **b** The photograph on the first day after surgery shows that the patient is able to cross his legs in lying position. **c** The photograph on the first day after surgery shows that the patient can cross his legs in sitting position. **d** The photograph on the first day after surgery shows that the patient is able to walk independently in a straight line in the absence of his feet external rotation

According to the patient's medical history, physical examination, and radiographic data, the patient was diagnosed to have GMC and moderate arthrokatadysis. In the past, most of patients with GMC and arthrokatadysis were treated with open surgery to achieve significantly improved gait and function. However, open surgery had many disadvantages, such as long incision, large trauma, much bleeding, some scar tissue hyperplasia and postoperative adhesion recurrence. With the development of sports medicine and the popularization of arthroscopy, some extra-articular orthopedic diseases could be treated by arthroscopy that is characteristic of insignificant trauma and bleeding, and early postoperative functional recovery. Nowadays, the release of gluteal contracture under arthroscopy is applied to treat GMC with good outcome[8]. Hence, we chose release of bilateral gluteus maximus contractures under arthroscopy for this patient. We used a three-portals technique near the greater trochanter of the hip joint (Fig. 2f). Two portals were generally applied to most patients, with one portal at the apex of the greater trochanter and the other at 2 cm or 4 cm above the apex of the greater trochanter, but in the current case with a severe GMC, an additional portal which was at 2 cm or 4 cm below the apex of the greater trochanter was used. We released the contracture of gluteal muscles, iliotibial band, and related fascia by radiofrequency under arthroscopy. The operation was conducted successfully with no adverse events or complications occurred. The postoperative incision was distributed as shown in the figure (Fig. 3a). On the first day after the operation, the patient could complete the cross-leg movement in bed (Fig. 3b). Although he was unable to place his right leg on his left leg completely, his left leg could easily cross the right leg in the sitting position, indicating the right side was more serious (Fig. 3c). The patient could walk in a straight line without external rotation of both feet (Fig. 3d).

## Discussion

Patients with arthrokatadysis present with the femoral head indentation that breaks through the wall of the acetabular wall and exceeds the Kohler's line, causing joint pain and restricted movement [9]. Arthrokatadysis is diagnosed by graphically measuring the vertical distance between the midpoint of the acetabular line (the medial wall of the acetabular wall) and the iliac line (the side of the quadrilateral). If the distance is ≥ 3 mm for an adult male or ≥ 6 mm for an adult female, arthrokatadysis can be diagnosed. Arthrokatadysis is classified into three degrees of severity according to the distance of acetabular inner edge beyond Kohler's line as follows: mild, 1–5 mm; moderate, 6–15 mm; severe, more than 15 mm. Arthrokatadysis is also attributable to numerous causative factors, such as rheumatoid arthritis, ankylosing spondylitis, psoriatic arthritis, reactive arthritis syndrome, acquired certificate of osteomalacia, osteoarthritis, hemophilia, post-traumatic invagination of the hip, osteogenesis imperfecta, septic arthritis, tumor, or radiation therapy to the destruction of the acetabulum, and etc. [10, 11]. In our case, we hypothesized that arthrokatadysis was caused by recurrent gluteal contracture fibrosis resulting from repeated bilateral buttocks injections. The study patient had moderate arthrokatadysis. Cases of GMC combined with pelvic tilt and hip dislocation have been reported in the literature [12], however, the current case with a severe GMC has not been seen before.

Considering the symptoms and signs of the current patient, we prescribed that he took a minimally invasive operation-arthroscopic surgical release of bilateral gluteus maximus contractures. The patient and his family were satisfied with the good outcome of the operation. Some scholars believe that GMC will not be reversed once it occurs, instead, it will gradually exacerbate [13]. If there is osteoarthritis of the hip joint or dislocation of the hip joint for a long time, total hip replacement should be performed [14, 15]. Although GMC was serious in this patient with a concurrent arthrokatadysis, no obvious signs of osteoarthritis and femoral head necrosis were found in both hips. Therefore, it was inappropriate to choose total hip replacement, as it is reported that arthroscopic surgery can remove the etiology of GMC [16, 17], resolve the patient's symptoms, and avoid further exacerbation of arthrokatadysis. The present outcome of the patient justified the therapy for the patient. The postoperative recovery of the patient was good and the symptoms were resolved significantly in the short-term follow-up. A long period of follow-up is necessary with focused attention to arthrokatadysis and femoral head morphological changes.

To summarize, this case report describes a patient with moderate arthrokatadysis caused by GMC due to long-term intramuscular injection of penicillin in the buttock, who underwent arthroscopic surgery and finally had good results. A delay in GMC diagnosis and intervention may incur arthrokatadysis to individuals affected.

## Data Availability

Not applicable.

## Abbreviations

GMC: Gluteal muscle contracture
ITB: Iliotibial band
CRP: C-reactive protein
RF: Rheumatoid factors
GPT: Glutamic pyruvic transaminase
GOT: Glutamic oxaloacetic transaminase
AKP: Alkaline phosphatase
γ-GT: Gamma glutamyl transpeptidase
THR: Total hip replacement
